# Application of Probabilistic Multiple-Bias Analyses to a Cohort- and a Case-Control Study on the Association between *Pandemrix*^™^and Narcolepsy

**DOI:** 10.1371/journal.pone.0149289

**Published:** 2016-02-22

**Authors:** Kaatje Bollaerts, Vivek Shinde, Gaël Dos Santos, Germano Ferreira, Vincent Bauchau, Catherine Cohet, Thomas Verstraeten

**Affiliations:** 1 P95 Pharmacovigilance and Epidemiology Services, Koning Leopold III Laan 1, 3001 Leuven, Belgium; 2 GSK Vaccines, 2301 Renaissance Boulevard, King of Prussia, PA 19406, United States of America; 3 Business & Decision Life Sciences (contractor for GSK Vaccines), Rue Saint Lambert 141, 1200 Brussels, Belgium; 4 GSK Vaccines, Avenue Fleming 20, B-1300 Wavre, Belgium; University of Hong Kong, HONG KONG

## Abstract

**Background:**

An increase in narcolepsy cases was observed in Finland and Sweden towards the end of the 2009 H1N1 influenza pandemic. Preliminary observational studies suggested a temporal link with the pandemic influenza vaccine *Pandemrix*™, leading to a number of additional studies across Europe. Given the public health urgency, these studies used readily available retrospective data from various sources. The potential for bias in such settings was generally acknowledged. Although generally advocated by key opinion leaders and international health authorities, no systematic quantitative assessment of the potential joint impact of biases was undertaken in any of these studies.

**Methods:**

We applied bias-level multiple-bias analyses to two of the published narcolepsy studies: a pediatric cohort study from Finland and a case-control study from France. In particular, we developed Monte Carlo simulation models to evaluate a potential cascade of biases, including confounding by age, by indication and by natural H1N1 infection, selection bias, disease- and exposure misclassification. All bias parameters were evidence-based to the extent possible.

**Results:**

Given the assumptions used for confounding, selection bias and misclassification, the Finnish rate ratio of 13.78 (95% CI: 5.72–28.11) reduced to a median value of 6.06 (2.5^th^- 97.5^th^ percentile: 2.49–15.1) and the French odds ratio of 5.43 (95% CI: 2.6–10.08) to 1.85 (2.5^th^—97.5^th^ percentile: 0.85–4.08).

**Conclusion:**

We illustrate multiple-bias analyses using two studies on the *Pandemrix*^™^-narcolepsy association and advocate their use to better understand the robustness of study findings. Based on our multiple-bias models, the observed *Pandemrix*^™^-narcolepsy association consistently persists in the Finnish study. For the French study, the results of our multiple-bias models were inconclusive.

## Introduction

In June 2009 the World Health Organization (WHO) declared an influenza pandemic phase 6 [1] caused by a novel influenza A/H1N1 strain leading the WHO’s Strategic Advisory Group of Experts recommending commercial-scale production of pandemic A(H1N1)pdm09 vaccines [2]. One of the vaccines authorized by the European Medicines Agency was *Pandemrix*^™^, an ASO3-adjuvanted, inactivated split-virion influenza virus A/California/7/2009 (H1N1)v-like strain vaccine (GSK Vaccines, Rixensart, Belgium). Worldwide, the vaccine was distributed in at least 47 countries, with over 30 million vaccinated individuals in Europe [3].

Early in 2010, an increase in narcolepsy cases was observed in children and adolescents in Finland and Sweden [4, 5], with preliminary investigations suggesting a temporal association with *Pandemrix*^™^, the only pandemic influenza vaccine used in these countries. Narcolepsy is a rare chronic sleep disorder, typically characterized by episodes of sudden loss of muscle tone, sometimes triggered by strong emotions (cataplexy) [6]. Narcolepsy etiology appears to be linked to genetic (HLA-DQB1*0602 allele [7]) and environmental (e.g., streptococcal infection [8], H1N1 natural infection [9, 10]) factors. To further assess the signal in Finland and Sweden, numerous observational studies at country level [11–19] were subsequently conducted in Europe and this in the best way possible under challenging circumstances (Table 1). Given the urgency of assessing the signal and the fact that the pandemic vaccine was no longer in use, all studies used retrospective designs combining data from various sources, thus potentially introducing systematic errors. The main sources of systematic errors that have been identified for the narcolepsy studies are ascertainment bias, disease onset misclassification, selection bias due to preferentially including exposed cases, diagnostic bias and confounding by indication and by natural H1N1 infection [20, 21]. Additionally, studies were affected by limited power as a result of the rarity of narcolepsy disease.

**Table 1:** Summary of observational single-country studies on narcolepsy following vaccination with *Pandemrix*^™^.

Country (region)	Vaccine Coverage	Design	Index date	Source	Exposure	Statistical analysis*	Study Period*	Study population: relative risk (95%CI) *
Sweden [18]	57.3%	Cohort	Diagnosis	Register	Register	HR: Cox regression	10/2009–12/2011	Children: 2.92 (1.78–4.79) Adults: 2.18 (1.00–4.75)
Sweden Stockholm [11]	52.6%	Cohort	Diagnosis	Register	Register	HR: Cox regression	10/2009–8/2010	Children: 1.54 (0.30–7.89) All ages: 1.45 (0.56–3.75)
Sweden (West) [19]	78%	Ecological	Diagnosis	Center/ Hospital		IRR: post- vs prevaccination	1/2000–12/2010	Children: 25 (N.A.)
Finland [16]	75%	Cohort	1stHCC	Register	Register	IRR: exposed vs unexposed	1/2009–8/2010	Children: 12.7 (6.1–30.8)
Finland [17]	75%	Ecological	Diagnosis	Register		IRR: post- vs prevaccination	2002–2010	All ages: 17 (N.A.)
Ireland [12]	20.8%	Cohort	1stHCC	Center/Hosp/GP	Temporary database	IRR: exposed vs unexposed	4/2008–12/2010	Children: 13.9 (5.2–37.2) Adults: 20.4 (1.8–225.0)
UK [15]	8%	Cases only	Symptom	Sleep Center	GP questionnaire	OR: logistic regression with offset	1/2008–7/2011	Children: 14.4 (4.3–48.5)
France [13]	8%	Case—Control	Diagnosis	Sleep Center	Self-report	OR: conditional logistic regression	10/2009–4/2011	Children: 6.5 (2.1–19.9) Adults: 4.7 (1.6–13.9)
Norway [14]	50%	Cohort	Symptom	Center/Hosp/GP	Register	IRR: exposed vs unexposed	10/2009–1/2010	Children: 10** (N.A.)

* Main analysis

** This is an approximation: The paper states ‘Our results showed….a minimum incidence of 10 out of 100,000 individuals in the vaccinated cohort…..This is an approximately 10-fold increase compared to the unvaccinated children’.

1stHCC = first health care contact, HR = hazard ratio, IRR = incidence rate ratio, OR = odds ratio

The use of quantitative bias analyses to study the impact of such systematic errors is advocated by key-opinion leaders [22–24] and international health authorities including regulatory agencies [25][26], especially for studies with far-reaching public health implications and leading to policy recommendations [22, 23]. This was not systematically done in any of the narcolepsy studies. In most studies, the potential bias assessment was limited to a qualitative discussion.

Several methods exist to quantify the impact of uncontrolled confounding [22, 24, 27–31] and bias [22, 24, 25, 27] on the risk estimates. These methods involve bias parameters, for which the values can be chosen to be either wide-ranging and hypothetical (‘Which bias levels are needed to reach a certain value?’: target-adjustment sensitivity analysis) or evidence-based (‘Which result would be obtained given a certain realistic level of bias?’: bias-level sensitivity analysis) [29]. Traditional deterministic bias analyses estimate the adjusted effect measure in light of the observed data and a finite set of bias parameter values. Such analyses can be improved upon using (probabilistic) Monte Carlo simulation, randomly sampling from the probability distributions of the bias parameters to obtain a distribution of the adjusted effect measure [31]. To jointly evaluate a cascade of different bias sources, multiple-bias analyses has been proposed [22, 24, 25, 27].

In this paper, we apply bias-level probabilistic multiple-bias analyses to two of the published narcolepsy studies in order to assess the robustness of the *Pandemrix*^™^ -narcolepsy association.

## Methods

### Selected studies

Multiple-bias analyses [22, 24, 25, 27] provide bias-adjusted estimates for effect measures derived from standard 2x2 tables. From the studies providing such effect measures [12–14, 16, 17, 19], we initially selected the Finnish cohort study because it has the strongest risk estimate (i.e. the highest lower limit of the 95% CI). To illustrate multiple-bias analyses for other designs, we also selected the French study, being the only case-control study (Table 1).

#### Finnish pediatric cohort study

This population-based study compared narcolepsy rates in a cohort of vaccinated to unvaccinated children aged 4–19 years [16] between 1999 and 2010. Vaccination status was obtained from primary health care databases. Narcolepsy cases (n = 67) were ascertained from the national care register and registers of three specialized health care centers following the Brighton Collaboration (BC) criteria [32], levels 1 to 3. The cohort vaccination coverage was 75%. For the primary analysis, the index date was the first documented health care contact and the follow-up period ended on August 15, 2010, the day before the media attention on post-vaccination narcolepsy started. The rate ratio (RR) was estimated at 12.7 (95% CI: 6.1–30.8).

#### French case-control study

A multi-center case-control study was carried out in 14 French expert orphan disease narcolepsy centers [13]. 59 cases (all ages) were recruited from patients with narcolepsy-cataplexy (BC Levels 1 and 2) [32], between 1 October 2009 and 30 April 2011. 135 controls were recruited from: (i) patients from the hospitals to which the participating sleep centers belonged; and (ii) healthy volunteers from a national database (Narcobank). Vaccination status was obtained through telephone interviews. The total population vaccination coverage was 8.8%. H1N1 vaccines used in France were *Pandemrix*^™^ (72%) and *Panenza*^™^ (28%). For the primary analysis, the index date was the date of polysomnography-MLST diagnosis. When considering all ages, the odds ratio (OR) for *Pandemrix*^™^ was 5.5 (95%CI: 2.5–12.0).

### Bias-level probabilistic multiple-bias analysis

For both studies, we built a Monte Carlo simulation model. The model structure consists of a sequence of bias correction equations [27] in the reverse order of the bias cascade [27, 29]. The model subsequently accounts for exposure misclassification, disease misclassification, selection bias and uncontrolled confounding by age, risk group and natural H1N1 infection (model structures in S1 Table) with evidence-based parameters. Tables 2 and 3 give an overview of the input distributions for the Finnish and French study, respectively. S2 and S3 Tables give details on the derivation of the input parameters. For every analysis, we generated 500.000 runs. All analyses were carried out in R 3.0.1 [33] (example code in S4 Table).

**Table 2:** Summary of Prior Distributions of the Monte Carlo based Multiple-bias Analyses, Finnish Pediatric Cohort Study (Nohynek, 2012)*.

Parameter	Description	Observations/distributions
Observed data		
n1	Number of vaccinated cases	46
n0	Number of unvaccinated cases	7
t1	Sum of follow-up time (person years) among vaccinated	510,874
t0	Sum of follow-up time (person years) among unvaccinated	986,195
Exposure misclassification		
SeX∣D = 1	Exposure sensitivity for cases	I(1)
SpX∣D = 1	Exposure specificity for cases	I(1)
SeX∣D = 0	Exposure sensitivity for non-cases	Bp(0.986,0.995,0.998)
SpX∣D = 0	Exposure specificity for non-cases	I(1)
Disease misclassification		
SeD∣X = 1^(1)^	Disease sensitivity for vaccinated	Bp(0.81,0.92,0.95)
FrD∣X = 1^(2)^	Number of false positive diagnoses per unit person-time among the vaccinated.	Bp(0.036,0.252,0.36)
SeD∣X = 0^(1)^	Disease sensitivity for unvaccinated.	Bp(0.28,0.34,0.79)
FrD∣X = 0^(2)^	Number of false positive diagnosis per unit person-time among unvaccinated.	Bp(0.0028,0.0084,0.028)
Selection bias		
	Negligible in this population-based cohort study	
Uncontrolled confounding: age group		
RR_CD(i)_	Association: age and narcolepsy	Bp(2.4,3.3,4.6)
PC∣X = 1_(i)_^(3)^	Prevalence age group 15–19yrs among vaccinated	I(0.28)
PC∣X = 0_(i)_^(3)^	Prevalence age group 15–19yrs among unvaccinated	I(0.56)
Uncontrolled confounding: risk group
RR_CD(ii)_	Association: risk group and narcolepsy	Bp(1.56,2.11,2.8)
PC∣X = 1_(ii)_^(4)^	Prevalence risk group among vaccinated	Bp(0.09,0.11,0.125)
PC∣X = 0_(ii)_^(4)^	Prevalence risk group among unvaccinated	Bp(0,0.04,0.09)
Uncontrolled confounding: natural H1N1 exposure
RR_CD(iii)_	Association: H1N1 infection and narcolepsy	Bp(14.9,16.4,17.5)
PC∣X = 1_(iii)_^(5)^	Prevalence H1N1 infection among vaccinated	Bp(0.29,0.30,0.32)
PC∣X = 0_(iii)_^(5)^	Prevalence H1N1 infection among unvaccinated	Bp(0.21,0.25,0.29)
Random error		
E	Random error for log(RR).	N(0,1/n1 + 1/n0)

* Details on the derivation of the input parameters are given in S2.

^(1)(2)(3) (4) (5)^ The parameters indicated with ^(.)^ are bivariately correlated with a correlation of 0.95. We used Gaussian copula’s to simulate correlated values.

Bp(min, mlik, max) = betapert distribution with a minimum, most likely, and maximum value; I(c) = deterministic distribution, a probability distribution of a random variable which only takes a single value c; N(μ, σ2) = normal distribution with mean μ and variance σ2; RR_CD_ = rate ratio between confounder and disease.

**Table 3:** Summary of Prior Distributions of the Monte Carlo Based Multiple-bias Analyses, French Case-control Study (Dauvilliers, 2013)*.

Parameter	Description	Observations/Distributions
Observed data		
A	Number of vaccinated cases.	31
B	Number of unvaccinated cases.	28
C	Number of vaccinated controls.	24
D	Number of unvaccinated controls.	111
Exposure misclassification		
SeX∣D = 1	Exposure sensitivity for cases.	I(1)
SpX∣D = 1	Exposure specificity for cases.	I(1)
SeX∣D = 0	Exposure sensitivity for controls.	Bp(0.97,0.98,1)
SpX∣D = 0	Exposure specificity for controls.	Bp(0.95,0.97,1)
Disease misclassification		
SeD∣X = 1	Disease sensitivity for vaccinated.	I(1)
SpD∣X = 1	Disease specificity for vaccinated.	I(1)
SeD∣X = 0	Disease sensitivity for unvaccinated.	I(1)
SpD∣X = 1	Disease specificity for unvaccinated.	I(1)
Selection bias		
Pcase∣X = 1	Selection probability of a vaccinated case	Pcase(a)∣X = 1 x Pcase(b)∣X = 1
	(a) reflecting differential ascertainment (Pcase(a)∣X = 1).	Bp(0.79,0.90,0.94)^(1)^
	(b) reflecting participation bias (Pcase(b)∣X = 1).	Bp(0.71,0.74,0.82)
Pcase∣X = 0	Selection probability of an unvaccinated case.	Pcase(a)∣X = 0 x Pcase(b)∣X = 0
	(a) reflecting differential ascertainment (Pcase(a)∣X = 0).	Bp(0.27,0.33,0.78)^(1)^
	(b) reflecting participation bias (Pcase(b)∣X = 0).	= b/(N/r—a/Pcase(b)∣X = 1)
Pcontrol∣X = 1	Selection probability of a vaccinated control.	= λ x Pcontrol∣X = 0, with
		λ ∼ Bp(0.8,1,1.2)
Pcontrol∣X = 0	Selection probability of an unvaccinated control.	I(C^t^)^+^
Uncontrolled confounding: age groups		
OR_CD(i)_	Association: age (18–29yrs vs. 5–17yrs) and narcolepsy	Bp(1.3,1.45,1.6)
PC∣X = 1_(i)_^(2)^	Prevalence age group 18–29yrs among vaccinated	Bp(0.13,0.15,0.17)
PC∣X = 0_(i)_^(2)^	Prevalence age group 18–29yrs among unvaccinated	Bp(0.22,0.24,0.26)
OR_CD(i)_	Association: age (30–50yrs vs. 5–17yrs) and narcolepsy	Bp(0.96,1.08,1.23)
PC∣X = 1_(i)_^(3)^	Prevalence age group 30–50yrs among vaccinated	Bp(0.45,0.47,0.49)
PC∣X = 0_(i)_^(3)^	Prevalence age group 30–50yrs among unvaccinated	Bp(0.43,0,44,0.46)
Uncontrolled confounding: risk group		
OR_CD_(i)	Association: ‘risk group’ and narcolepsy.	Bp(1.56,2.11,2.8)
PC∣X = 1(i)^(4)^	Prevalence of ‘risk group’ among vaccinated.	Bp(0.15,0.21,0.26)
PC∣X = 0(i)^(4)^	Prevalence of ‘risk group’ among unvaccinated.	Bp(0.1,0.11,0.12)
Uncontrolled confounding: natural H1N1 exposure		
OR_CD_(ii)	Marginal association: H1N1 infection and narcolepsy.	Bp(14.9,16.4,17.5)
PC∣X = 1(ii)^(5)^	Prevalence of H1N1 infection among vaccinated.	Bp(0.29,0.34,0.42)
PC∣X = 0(ii)^(5)^	Prevalence of H1N1 infection among unvaccinated.	Bp(0.28,0.285,0.29)
Random error		
E	Random error for log(OR).	N(0,1/a + 1/b + 1/c + 1/d)

* Details on the derivation of the input parameters are given in S3.

^(1)(2)(3) (4) (5)^ The parameters indicated with ^(.)^ are bivariately correlated with a correlation of 0.95. We used Gaussian copula’s to simulate correlated values.

Bp(min, mlik, max) = Betapert distribution with minimum, most likely and maximum value.

I(c) = Degenerate distribution, a probability distribution of a random variable which only takes a single value c.

N(μ, σ2) = Normal distribution with mean μ and variance σ2.

OR_CD_ = odds ratio between confounder and disease.

^+^ Because only disproportionality in sampling weights causes selection bias, the constant C^t^ can take any value.

### Input parameters: Finnish pediatric cohort study

#### Exposure misclassification

We judged that recall bias was negligible as the index date was the first reported health care contact. The authors assessed incompleteness of the exposure data through reviewing the local vaccination records of 1000 randomly selected individuals from the study cohort [16]. This review revealed four discrepancies, all of whom had been vaccinated but were not recorded as such, implying an exposure sensitivity for non-cases (Se_X∣D = 0_) of 0.995 given the cohort vaccination coverage of 75% [16]. To account for parameter uncertainty, Se_X∣D = 0_ was assumed to follow a betapert distribution [34] A betapert distribution is a continuous, smooth, unimodal distribution described by a minimum (min.), most likely (mlik.) and maximum (max.) value and which is commonly used in risk analysis to reflect expert opinion or in situations with limited data. As the review did not reveal false positives, the exposure specificity for non-cases was assumed perfect (Sp_X∣D = 0_ = 1). The review of the vaccination records of all narcolepsy cases did not show any discrepancies, implying perfect exposure sensitivity and specificity among cases (Se_X∣D = 1_ = Sp_X∣D = 1_ = 1).

#### Disease misclassification

Differential ascertainment and lack of blinding are two potential sources of disease misclassification. Differential ascertainment bias occurs if vaccinated cases are (1) more likely to seek health care, (2) more likely to be referred to a specialized center and/or (3) more likely to be diagnosed compared to unvaccinated cases. These factors act multiplicatively, implying large potential for differential ascertainment bias. Indirect evidence for differential ascertainment can be found in the Swedish studies, showing a decreasing risk estimate from 4.19 (95% CI: 1.76, 12.1) to 2.92 (95% CI: 1.78, 4.79) with one additional year of follow-up [18, 35], likely explained by the increasing narcolepsy incidence in the unvaccinated cohort with the extended follow-up [18]. Additional evidence is the shorter time-to-diagnosis among vaccinated (12 months, 7.9 months, 6.9 months) compared to unvaccinated subjects (60 months, 47.6 months, 12.6 months) as observed in the French [13, 17, 19]. We obtained the disease sensitivity among the vaccinated (Se_D∣X = 1_) and unvaccinated (Se_D∣X = 0_) starting from the observed time-to-diagnosis intervals and assuming an exponential distribution for time. We further assumed that the disease sensitivities are highly correlated with a correlation of 0.95 and used Gaussian copula’s to simulate correlated values.

Lack of blinding among the referred potential cases may result in a higher false positive probability among vaccinated compared to unvaccinated subjects. In the Finnish cohort study [16], narcolepsy cases were classified with increasing uncertainty as BC level 1 (16%), level 2 (76%) and level 3 (8%). We assume that false positives occur among level 3 cases only. The differential false positivity rate due to lack of blinding was reflected by assuming that the proportion of false positives among the BC level 3 cases was higher in vaccinated (min. = 5%, mlik. = 35%, max. = 50%) compared to unvaccinated subjects (min. = 5%, mlik. = 15%, max. = 50%). We also assumed a correlation of 0.95 for the false positive rates.

#### Selection bias

Selection bias is negligible in this population-based cohort study because the Finnish computerized national register was used. In this register, all residents are recorded and assigned a personal identity code that remains unchanged throughout a person’s lifetime.

#### Uncontrolled confounding

Confounding by age, by indication and by natural H1N1 infection are the most important recognized uncontrolled confounders. To correct for uncontrolled confounding, assumptions are needed about (i) the association between the confounder and the disease (RR_CD_), (ii) the prevalence of the confounder among the vaccinated (P_C∣X = 1_) and (iii) among the unvaccinated subjects (P_C∣X = 0_) [24].

Age might have acted as a confounder because the typical narcolepsy onset is between 10–30 years of life and the vaccination coverage was different by age (5–9 years: 80.6%; 10–14 years: 81,9%15–19 years: 56,6%) [16]. For the bias analyses, we used the age groups 5–14 years and 15–19 years and derived the confounder-disease association (RR_CD_) from the age-specific narcolepsy rates for the period 2000–2010 as obtained from a large European study on the incidence of narcolepsy [36]. The prevalence of the confounder (i.e. the age group 15–19 years) within the vaccinated (P_C∣X = 1_) and unvaccinated Finnish population (P_C∣X = 0_) was derived from the age-specific Pandemrix vaccination coverage [16].

Confounding by indication would occur if belonging to a particular risk group for which H1N1 vaccination was recommended also carried an increased risk to develop narcolepsy. We derived the confounder-disease association (RR_CD_) from the British narcolepsy study [15], which reported the odds ratio with and without matching on risk group. Then, we estimated the distribution of ‘risk group’ within the vaccinated (P_C∣X = 1_) and unvaccinated Finnish population (P_C∣X = 0_) by assuming the vaccination coverage within the risk group being minimally the overall vaccination coverage of 75%.

Confounding by natural H1N1 infection would occur if individuals exposed to natural H1N1 infection prior to protection through vaccination would be more likely to get vaccinated (being more likely to seek care and be offered vaccination) compared to non-exposed individuals. We derived the confounder-disease association from a Chinese study, which showed strong seasonality in narcolepsy occurrences correlated with both H1N1 and seasonal influenza counts assuming a partial population exposure of 31.8% (95%CI: 29.1–34.1) as observed in a Chinese serological study among children (6–15yrs) [37]. We then calculated the prevalence of H1N1 infection among the vaccinated (P_C∣X = 1_) and unvaccinated (P_C∣X = 0_) Finnish population given the Finnish vaccination coverage of 75% while assuming an H1N1 attack rate of 29% (95% CI: 15.8–41.9) [38] and a relative risk of vaccination given H1N1 exposure of minimum 1.0, most likely 1.2 and maximum 1.5. Hereby, we assume, as in [39], that vaccination effectively took place after the first H1N1 season as the H1N1 vaccination campaign for the healthy population in Finland started a week after the peak epidemic in the country [40].

For each of the three recognized sources of uncontrolled confounding, we assumed a bivariate correlation of 0.95 between the confounder prevalence rates within the vaccinated and unvaccinated population.

### Input parameters: French case-control study

#### Exposure misclassification

The self-reported vaccination status used within the study might induce recall bias [13]. For all cases, vaccination was documented, implying perfect exposure specificity (Sp_X∣D = 1_). We assumed perfect exposure sensitivity (Se_X∣D = 1_) since medical records of cases were scrutinized. For six controls, vaccination could not be documented [13]. We calculated the exposure specificity (Sp_X∣D = 0_) assuming that all, three and none of the ‘undocumented’ controls were false positive. Exposure sensitivity among controls (Se_X∣D = 1_) was assumed to be high, but not perfect. In studies comparing self-reported influenza vaccination status to vaccination records, the average exposure sensitivity is 96% [41]. Because a mass vaccination campaign was implemented in France during the H1N1 influenza period [42], the exposure sensitivity was assumed to be higher than 96%.

#### Disease misclassification

As only BC level 1 and 2 cases were retained, disease misclassification was assumed negligible.

#### Selection bias

Differential ascertainment (diagnosis is necessary for case selection) and participation bias potentially affect the selection probability of a case. Similar to the Finnish study and starting from the observed time-to-diagnosis intervals, we obtained the selection probability referring to the ascertainment of a vaccinated (P_case(a)∣X = 1_) and unvaccinated case (P_case(a)∣X = 0_). We obtained the selection probability due to participation for vaccinated (P_case(b)∣X = 1_) and unvaccinated cases (P_case(b)∣X = 0_) starting from the number of participating cases (n = 59) and the participation rate (71%). We assumed that exposure prevalence among the non-participating cases was minimum 30%, most likely 45% and maximum 59% (with 59% being the exposure prevalence among participating cases). As ascertainment bias and participation bias are independent given exposure, the overall sampling probability is the product of the bias-specific sampling probabilities. We further assumed that the selection probabilities related to the ascertainment of vaccinated and unvaccinated cases were highly correlated with a correlation of 0.95.

The selection probability of controls was judged to be subject to substantial uncertainty because (i) the participation rate among controls was not given, (ii) hospital controls were recruited from departments that specialized in treatment of patients for which H1N1-vaccination was not recommended and (iii) healthy controls were recruited from NARCOBANK, among which many healthcare workers for which vaccination was recommended [43]. Hence, the selection probability was unlikely to be independent of exposure with bias in either direction. Therefor, we assumed the ratio of the selection probabilities of vaccinated and unvaccinated controls (P_control∣X = 1_/ P_control∣X = 0_) to be minimum 0.8, most likely 1 and maximum 1.2.

#### Uncontrolled confounding

To adjust for confounding by age, we used the age groups 5–17 years, 18–29 years and 30–50 years having a H1N1 vaccination coverage of +/- 10%, 4% and 7,5% [42]. Similarly as for the Finnish study, we derived the confounder-disease associations from the age-specific narcolepsy rates obtained from a large European study [36]. The prevalence rates of the polytomous confounder within the vaccinated and unvaccinated French population were derived from the age-specific H1N1 influenza vaccination coverage [16].

For confounding by indication, we made the same assumption for the confounder-disease association (OR_CD_) as for the Finnish study and estimated the distribution of ‘risk group’ within the vaccinated (P_C∣X = 1_) and unvaccinated French population (P_C∣X = 0_) based on the French coverage data (risk group = 21%; non-risk group = 12%) [44].

Also for confounding by natural H1N1 infection, we made the similar assumptions as for the Finnish cohort study. We calculated the prevalence of H1N1 infection starting from the overall vaccination coverage in France (8.8%) and assuming a relative risk of vaccination given H1N1 exposure of minimum 1, most likely 1.2 and maximum 1.5.

Again, for each of the three recognized sources of uncontrolled confounding, we assumed a bivariate correlation of 0.95 between the confounder prevalence rates within the vaccinated and unvaccinated population.

## Results

### Finnish pediatric cohort study

Given the assumptions made, we found disease misclassification (as a result of differential ascertainment) to have the largest decreasing impact on the rate ratio and confounding by age to have the largest increasing impact (Tables 4 and 5). Combining all recognized sources of bias and uncontrolled confounding, the original rate ratio of 13.78 (95% CI: 5.72–28.11) reduced to a median value of 6.06 (2.5^th^ -97.5^th^ percentile: 2.49–15.1).

**Table 4:** Results of the Monte Carlo Based Multiple-bias Analyses of the Finnish Pediatric Cohort Study [16] and the French Case-Control Study [13].

	study	Finnish cohort (Rate Ratio)			French case-control (Odds Ratio)		
Bias model	percentile	50^th^	2.5^th^,	97.5^th^	50^th^	2.5^th^,	97.5^th^
No bias adjustment^+^		12.67	5.73	28.17	5.12	2.61	10.06
Exposure misclassification		12.57	5.69	27.9	5.97	3.01	11.82
Disease misclassification		5.48	2.33	13.15			
Selection bias					1.94	0.9	4.26
Confounding: age group		17.7	8	39.32	5.3	2.69	10.44
Confounding: risk group		11.84	5.34	26.23	4.66	2.37	9.16
Confounding: H1N1 infection		10.87	4.89	24.16	4.38	2.21	8.65
Exposure misclassification, disease misclassification		5.44	2.31	12.96			
Exposure misclassification, selection bias					2.26	1.04	5.02
Exposure misclassification, confounding by age		17.54	7.9	38.84	6.17	3.1	12.28
Exposure misclassification, confounding by risk group		11.73	5.29	26	5.43	2.73	10.83
Exposure misclassification, confounding by H1N1 infection		10.8	4.85	23.97	5.11	2.54	10.24
Disease misclassification, confounding by age		7.65	3.25	18.42			
Disease misclassification, confounding by risk group		5.11	2.16	12.4			
Disease misclassification, confounding by H1N1 infection		4.7	1.96	11.58			
Selection bias, confounding by age					2.01	0.93	4.41
Selection bias, confounding by risk group					1.77	0.83	3.83
Selection bias, confounding by H1H1 infection					1.66	0.79	3.51
Exposure-, disease misclassification, confounding by age		7.58	3.22	18.2			

^+^ Estimates based on the Monte Carlo simulation model, closely approximating the reported estimates

**Table 5:** Results of the Monte Carlo Based Multiple-bias Analyses of the Finnish Pediatric Cohort Study [16] and the French Case-Control Study [13], continued.

	study	Finnish cohort (Rate Ratio)			French case-control (Odds Ratio)		
Bias model	percentile	50^th^	2.5^th^,	97.5^th^	50^th^	2.5^th^,	97.5^th^
Exposure-, disease misclassification, confounding by risk group		5.06	2.14	12.21			
Exposure-, disease misclassification, confounding by H1H1 infection		4.66	1.94	11.47			
Exposure-, disease misclassification, confounding by risk group and H1N1 infection		4.35	1.8	10.8			
Exposure-, disease misclassification, confounding by age, risk group and H1N1 infection		6.06	2.49	15.1			
Exposure misclassification, selection bias, confounding by age					2.34	1.08	5.2
Exposure misclassification, selection bias, confounding by risk group					2.06	0.96	4.51
Exposure misclassification, selection bias, confounding by H1N1 infection					1.94	0.92	4.12
Exposure misclassification, selection bias, confounding by risk group and H1N1 infection					1.76	0.84	3.72
Exposure misclassification, selection bias, confounding by age, risk group and H1N1 infection					1.82	0.83	4.07

### French case-control study

In this study, we found selection bias (as a result of differential ascertainment, participation bias and improper selection of controls) to have the largest impact on the odds ratio (Tables 4 and 5), yielding an adjusted odds ratio with a median value of 1.94 (2.5^th^ -97.5^th^ percentile: 0.9–4.26). Combining all recognized sources of bias and confounding, the original odds ratio of 5.43 (95% CI: 2.6–10.08) reduced to a median value of 1.82 (2.5^th^ -97.5^th^ percentile: 0.83–4.07), with the inter-percentile range including 1.

## Discussion

We performed multiple-bias analyses on the Finnish [16] and French [13] narcolepsy studies using Monte Carlo simulation models with evidence-based distributions for the bias parameters. We are not aware that such systematic bias analyses embedded within formal models of bias and confounding were performed in any of the *Pandemrix*^™^-narcolepsy studies. Most sensitivity analyses reported so far assessed how certain operational study choices affected the results, such as using different onset dates [12, 13, 15, 16], different follow-up periods [12, 13, 15, 16] or different inclusion criteria [13, 15]. Only in the UK study [15], deterministic single-bias analyses on case selection and confounder misclassification were carried out. In the Irish study [12], one deterministic bias analysis on case selection bias was performed. Although the risk estimates substantially decreased, they remained significant in these single-bias analyses.

In our multiple-bias analyses and given our base case assumptions, the *Pandemrix*^™^-narcolepsy association consistently persists in the Finnish study; The rate ratio of the Finnish study reduced from 12.7 to 6.06 (2.5^th^-97.5^th^ percentile: 2.49–15.1) after adjusting for the recognized bias sources. The risk estimates of the French study proved to be less robust; Given our base case assumptions for all recognized bias source, the odds ratio decreased to 1.85 (2.5^th^-97.5^th^ percentile: 0.85–4.08), with the inter-percentile range including 1.

Ascertainment bias (i.e. disease misclassification in a population-based cohort and selection bias in a case-control study) was found to have the largest decreasing effect on the risk estimates. This large impact might be explained by the characteristics of narcolepsy, being rare under diagnosed disorder with often an insidious onset characterized by a very long interval from first symptoms to final diagnosis (up to 15 years on average [45]), in combination with a relatively short study period.

Our approach has limitations. We only quantified the impact of the recognized sources of bias and confounding, though other, so far unrecognized sources might exist. The assumptions were evidence-based to the extent possible. Nevertheless some assumptions were based on limited information, in particular the assumption on the H1N1 infection-narcolepsy association or the differential H1N1 attack rate among vaccinated and unvaccinated. We further assumed that the shortened time-to-diagnosis among the vaccinated cases [13, 17, 19] was an indication of ascertainment bias. This difference could alternatively be explained by a more severe clinical presentation. However, the comparison of other disease characteristics, such as hypocretin levels or sleep latency test results, between vaccinated and unvaccinated cases does not support this [17, 19, 43, 46, 47]. Another explanation for the differential time to diagnosis in [13, 17, 19] could be the differential observation periods, whereby unvaccinated cases could have symptom onset before the pandemic, but vaccinated cases could only have symptom onset started as of the pandemic. To assess this, we used an alternative parametrization of ascertainment bias, based on the Swedish observation that the narcolepsy incidence among unvaccinated more than doubled after public awareness [18]. This approach resulted in smaller reduction of the risk estimates with the association now consistently persisting for the Finnish and French study, with a median RR of 7.48 (2.5^th^ -97.5^th^ percentiles: 3.3–16.9) for the Finnish study and median OR of 2.26 (2.5^th^ -97.5^th^ percentiles: 1.09–4.67) for the French study when adjusting for all considered sources of bias.

Finally, it needs to be stressed that multiple-bias analyses assess the sensitivity of the observed risk estimate to hypothetical values of plausible sources of bias. They do not assess the presence of a specific source of bias, nor do they provide a true estimate of the causal association. Keeping in mind that conventional methods rely on the implicit assumption of no bias and no confounding, bias-analysis are a good quantitative extension of the qualitative speculations on bias and confounding that characterize a good discussion of study results [48]. We provided details on the derivation of the input parameters (S2 and S3 Tables) and example code (see S4 Table). This way, the analyses are fully transparent and reproducible and can easily be rerun using different distributions for the bias parameters.

We performed our analyses on two of the published *Pandemrix*^™^-narcolepsy studies and found that the association consistently persists in the Finnish study but not in the French study. We encourage the authors of the narcolepsy studies to take potential measured confounders into account as much as possible in their original statistical analyses and to perform multiple-bias analyses for these sources of bias and confounding that cannot be adjusted for in the original analyses. Whether the results reported in this paper change the conclusions of the *Pandemrix*^™^-narcolepsy association is a matter of debate. At the very least, more detailed sensitivity analyses allow a better understanding of the sensitivity of each study’s finding to such presumed biases, which may eventually lead to more informed benefit-risk and public health decision-making.

### Trademark statement

*Pandemrix* is a trademark of GSK group of companies.

## Supporting Information

S1 Table Bias correction equations, cohort study and case-control study. (DOCX)

S2 Table Input parameters Finnish cohort study, detailed. (DOCX)

S3 Table Input parameters French case-control, detailed. (DOCX)

S4 Table Example code in R. (DOCX)
