# Supplementary material for: Application of Probabilistic Multiple-Bias Analyses to a Cohort- and a Case-Control Study on the Association between Pandemrix™and Narcolepsy
Source: PLoS One. 2016 Feb 22;11(2):e0149289. doi: 10.1371/journal.pone.0149289 (PMC4762678; doi:10.1371/journal.pone.0149289)
Supplement: S1 Table — (DOCX) [file pone.0149289.s001.docx]

S1. Multiple-bias correction equations in the reverse order of the potential bias cascade.

Cohort study^*^

|  |  |  |  |
| --- | --- | --- | --- |
| **Step 1: Exposure misclassification** | | | |
|  | -Number of exposed cases ‘adjusted’ for exposure misclassification. | | |
|  | n_1(1)_ = [(Sp_X∣D=1_ x n_1_)-((1- Sp_X∣D=1_) x n_0_)] / [( Se_X∣D=1_ x Sp_X∣D=1_) - ((1- Se_X∣D=1_ ) x (1- Sp_X∣D=1_))], | | |
|  |  | n_0_ = number of exposed cases, n_1_ = number of unexposed cases, | |
|  |  | Se_X∣D=1_ = exposure sensitivity for cases, Sp_X∣D=1_ = exposure specificity for cases. | |
|  | -Number of unexposed cases ‘adjusted’ for exposure misclassification. | | |
|  | n_0(1)_ = n - n_1(1)_ , | | |
|  |  | n = number of cases (n_0_ + n_1_). | |
|  | -Follow-up time (person years) among exposed ‘adjusted’ for exposure misclassification. | | |
|  | t_1(1)_ = [(Sp_X∣D=0_ x t_1_)-((1- Sp_X∣D=0_ ) x t_0_)] / [(Se_X∣D=0_ x Sp_X∣D=0_ ) - ((1- Se_X∣D=0_ ) x (1- Sp_X∣D=0_ ))], | | |
|  |  | t_1_ = follow-up time (person years) among exposed, t_0_ = follow-up time (person years) among unexposed, | |
|  |  | Se_X∣D=0_ = exposure sensitivity for non-cases, Sp_X∣D=0_ = exposure specificity for non-cases. | |
|  | -Follow-up time (person years) among unexposed ‘adjusted’ for exposure misclassification. | | |
|  | t_0(1)_ = t - t_1(1)_, | | |
|  |  | t = total amount of follow-up time (t_0_ + t_1_). | |
|  | -Rate ratio ‘adjusted’ for exposure misclassification. | | |
|  | RR_(1)_= (n_1(1)_/ t_1(1)_)/(n_0(1)_/ t_0(1)_). | | |
|  |  |  | |
| **Step 2: Disease misclassification** | | | |
|  | -Number of exposed cases ‘adjusted’ for exposure and disease misclassification. | | |
|  | n_1(2)_ = (n_1(1)_ - Fr_D∣X=1_ x t_1(1)_)/Se_D∣X=1_, | | |
|  |  | Se_D∣X=1_ = disease sensitivity for exposed, | |
|  |  | Fr_D∣X=1_ = number of false positive diagnoses per unit person-time among the exposed. | |
|  | -Number of unexposed cases ‘adjusted’ for exposure disease misclassification. | | |
|  | n_0(2)_ = (n_0(1)_ - Fr_D∣X=0_ x t_0(1)_)/Se_D∣X=0_, | | |
|  |  | Se_D∣X=0_ = disease sensitivity for unexposed, | |
|  |  | Fr_D∣X=0_= number of false positive diagnoses per unit person-time among the unexposed. | |
|  | -Follow-up time (person years) among exposed ‘adjusted’ for exposure and disease misclassification. | | |
|  | t_1(2)_ = t_1(1)_.^**^ | | |
|  | -Follow-up time (person years) among unexposed ‘adjusted’ for exposure and disease misclassification. | | |
|  | t_0(2)_ = t_0(1)_.^**^ | | |
|  | -Rate ratio ‘adjusted’ for exposure and disease misclassification. | | |
|  | RR_(2)_= (n_1(2)_/ t_1(2)_)/(n_0(2)_/ t_0(2)_). | | |
|  |  |  | |
| **Step 3: Selection bias** | | | |
|  | -Number of exposed cases ‘adjusted’ for exposure-, disease misclassification and selection bias. | | |
|  | n_1(3)_ = n_1(2)_/p_select∣D=1,X=1_, | | |
|  |  | p_select∣D=1,X=1_ = selection probability of a exposed case. | |
|  | -Number of unexposed cases ‘adjusted’ for exposure-, disease misclassification and selection bias. | | |
|  | n_0(3)_ = n_0(2)_/p_select∣D=1,X=0_ , | | |
|  |  | p_select∣D=1,X=0_ = selection probability of a unexposed case. | |
|  | -Follow-up time (person years) among exposed ‘adjusted’ for exposure-, disease misclassification and selection bias. | | |
|  | t_1(3)_ = t_1(2)_/p_select∣X=1_,  _,,,_,  , | | |
|  |  | p_select∣X=1_ = selection probability of exposed. | |
|  | -Follow-up time (person years) among unexposed ‘adjusted’ for exposure-, disease misclassification and selection bias. | | |
|  | t_0(3)_ = t_0(2)_/p_select∣X=0_, | | |
|  |  | p_select∣X=0_ = selection probability of unexposed. | |
|  | -Rate ratio ‘adjusted’ for exposure-, disease misclassification and selection bias. | | |
|  | RR_(3)_= (n_1(3)_/ t_1(3)_)/(n_0(3)_/ t_0(3)_). | | |
|  | RR_(3)_= (n_1(3)_/ t_1(3)_)/(n_0(3)_/ t_0(3)_). | | |

| **Step 4: ‘Unmeasured’ confounding** | | |
| --- | --- | --- |
| -Rate ratio ‘adjusted’ for exposure-, disease misclassification, selection bias and ‘unmeasured’ confounding. | | |
| RR_(4)_ = RR_(3)_ [RR_CD_ P_C∣X=0_ + (1- P_C∣X=0_ )] /[ RR_CD_ P_C∣X=1_ + (1- P_C∣X=1_ )],**^***^** | | |
|  | RR_CD_ = marginal association between confounder and disease, P_C∣X=1_ = prevalence of confounder among exposed, | |
|  | P_C∣X=0_ = prevalence of confounder among unexposed. | |
|  |  | |
| **Step 5: Random error** | | |
| -RR_(5)_ = exp(ln(RR_(4)_)+e), | |  |
|  | e = random error. |  |
| ^*^ The correction equations are based on Greenland (1996), Basic methods for sensitivity analysis of biases. *IJE*, 26,1107-1116. ^**^Unless the disease is very common, false negatives add little person-time and t_1(2)_ is approximated by t_1(1)_. ^***^ This equation can be applied recursively to account for several sources of ‘unmeasured’ confounding (Schlesselman 1978, Assessing effects of confounding variables, *AJE*, 108:3-8). | |  |

Case-control study^*^

|  |  |  |  |
| --- | --- | --- | --- |
| **Step 1: Exposure misclassification** | | | |
|  | -Number of exposed cases ‘adjusted’ for exposure misclassification. | | |
|  | a_(1)_ = [(Sp_X∣D=1_ x a)-((1- Sp_X∣D=1_) x b)] / [( Se_X∣D=1_ x Sp_X∣D=1_) - ((1- Se_X∣D=1_ ) x (1- Sp_X∣D=1_)], | | |
|  |  | a = number of exposed cases, b = number of unexposed cases, | |
|  |  | Se_X∣D=1_ = exposure sensitivity for cases, Sp_X∣D=1_ = exposure specificity for cases. | |
|  | -Number of unexposed cases ‘adjusted’ for exposure misclassification. | | |
|  | b_(1)_ = n - a_(1)_ , | | |
|  |  | n = number of cases (a + b). | |
|  | - Number of exposed controls ‘adjusted’ for exposure misclassification. | | |
|  | c_(1)_ = [(Sp_X∣D=0_ x c)-((1- Sp_X∣D=0_ ) x d)] / [(Se_X∣D=0_ x Sp_X∣D=0_ ) - ((1- Se_X∣D=0_ ) x (1- Sp_X∣D=0_ ))], | | |
|  |  | c = number of exposed controls, d = number of unexposed controls, | |
|  |  | Se_X∣D=0_ = exposure sensitivity for non-cases, Sp_X∣D=0_ = exposure specificity for non-cases. | |
|  | - Number of unexposed controls ‘adjusted’ for exposure misclassification. | | |
|  | d_(1)_ = m - c_(1)_, | | |
|  |  | m = number of controls (c + d). | |
|  | -Odds ratio ‘adjusted’ for exposure misclassification. | | |
|  | OR_(1)_ = (a_(1)_ x d_(1)_)/(b_(1)_ x c_(1)_) | | |
|  |  |  | |
| **Step 2: Disease misclassification** | | | |
|  | -Number of exposed cases ‘adjusted’ for exposure and disease misclassification. | | |
|  | a_(2)_ = [a_(1)_ - (a_(1)_ + c_(1)_) x (1- Sp_D∣X=1_)] / [Se_D∣X=1_ – (1- Sp_D∣X=1_ )] , | | |
|  |  | Se_D∣X=1_ = disease sensitivity for exposed, Sp_D∣X=1_ = disease specificity for exposed. | |
|  | -Number of unexposed cases ‘adjusted’ for exposure and disease misclassification. | | |
|  | b_(2)_ = [b_(1)_ - (b_(1)_ + d_(1)_) x (1- Sp_D∣X=0_)] / [Se_D∣X=0_ – (1- Sp_D∣X=0_ )] , | | |
|  |  | Se_D∣X=0_ = disease sensitivity for unexposed, Sp_D∣X=0_ = disease specificity for unexposed. | |
|  | - Number of exposed controls ‘adjusted’ for exposure and disease misclassification. | | |
|  | c_(2)_ = (a_(1)_ +c_(1)_) - a_(2)_. | | |
|  | - Number of unexposed controls ‘adjusted’ for exposure and disease misclassification. | | |
|  | d_(2)_ = (b_(1)_ +d_(1)_) - b_(2)_. | | |
|  | - Odds ratio ‘adjusted’ for exposure and disease misclassification. | | |
|  | OR_(2)_ = (a_(2)_ x d_(2)_)/(b_(2)_ x c_(2)_). | | |
|  |  |  | |
| **Step 3: Selection bias** | | | |
|  | -Number of exposed cases ‘adjusted’ for exposure-, disease misclassification and selection bias. | | |
|  | a_(3)_ = a_(2)_/p_select∣D=1,X=1_, | | |
|  |  | p_select∣D=1,X=1_ = selection probability of a exposed case. | |
|  | -Number of unexposed cases ‘adjusted’ for exposure-, disease misclassification and selection bias. | | |
|  | b_(3)_ = b_(2)_/p_select∣D=1,X=0_, | | |
|  |  | p_select∣D=1,X=0_ = selection probability of a unexposed case. | |
|  | - Number of exposed controls ‘adjusted’ for exposure-, disease misclassification and selection bias. | | |
|  | c_(3)_ = c_(2)_/p_select∣D=0,X=1_, | | |
|  |  | p_select∣D=0,X=1_ = selection probability of a exposed control. | |
|  | - Number of unexposed controls ‘adjusted’ for exposure-, disease misclassification and selection bias. | | |
|  | d_(3)_ = d_(2)_/p_select∣D=0,X=0_, | | |
|  |  | p_select∣D=0,X=0_ = selection probability of a unexposed control. | |
|  | -Odds ratio ‘adjusted’ for exposure-, disease misclassification and selection bias. | | |
|  | OR_(3)_ = (a_(3)_ x d_(3)_)/(b_(3)_ x c_(3)_). | | |
|  |  |  | |
| **Step 4: ‘Unmeasured’ confounding** | | | |
|  | -Odds ratio ‘adjusted’ for exposure-, disease misclassification, selection bias and ‘unmeasured’ confounding. | | |
|  | OR_(4)_ = OR_(3)_ [OR_CD_ P_C∣X=0_ + (1- P_C∣X=0_ )] /[ OR_CD_ P_C∣X=1_ + (1- P_C∣X=1_ )], **^**^** | | |
|  |  | OR_CD_ = marginal association between confounder and disease, P_C∣X=1_ = prevalence of confounder among exposed, | |
|  |  | P_C∣X=0_ = prevalence of confounder among unexposed. | |
|  |  |  | |
| **Step 5: Random error** | | | |
|  | -OR_(5)_ = exp(ln(OR_(4)_)+e), | | |
|  |  | e = random error. | |
|  | ^*^ The correction equations are based on Greenland (1996), Basic methods for sensitivity analysis of biases. *IJE*, 26,1107-1116. ^**^ This equation can be applied recursively to account for several sources of ‘unmeasured’ confounding (Schlesselman 1978, Assessing effects of confounding variables, *AJE*, 108:3-8). | | |
