# Supplementary material for: Application of Probabilistic Multiple-Bias Analyses to a Cohort- and a Case-Control Study on the Association between Pandemrix™and Narcolepsy
Source: PLoS One. 2016 Feb 22;11(2):e0149289. doi: 10.1371/journal.pone.0149289 (PMC4762678; doi:10.1371/journal.pone.0149289)
Supplement: S2 Table — (DOCX) [file pone.0149289.s002.docx]

S2 Derivation of prior distributions for the Monte Carlo based multiple-bias analyses, Finnish pediatric cohort-study (Nohynek, 2012).

Part A: Observed data

| Parameter | | Description: Rationale | Distribution |
| --- | --- | --- | --- |
|  |  |  |  |
| Observed data | | | |
|  | n_1_ | Number of vaccinated cases. | 46 |
|  | n_0_ | Number of unvaccinated cases. | 7 |
|  | t_1_ | Follow-up time (person years) among vaccinated. | 510,874 |
|  | t_0_ | Follow-up time (person years) among unvaccinated. | 986,195 |

S2 Derivation of prior distributions for the Monte Carlo based multiple-bias analyses, Finnish pediatric cohort-study (Nohynek, 2012).

Part B: Exposure misclassification

| Parameter | | | Description: Rationale | Distribution  (min,mlik,max) | | |
| --- | --- | --- | --- | --- | --- | --- |
|  |  | |  |  | | |
| Exposure misclassification | | | | | | |
| Se_X∣D=1_ | | Exposure sensitivity for cases: The review of the vaccination records of all narcolepsy cases did not reveal any discrepancy (12). | | | I(1) |  |
| Sp_X∣D=1_ | | Exposure specificity for cases: The review of the vaccination records of all narcolepsy cases did not reveal any discrepancy (12). | | | I(1) |  |
| Se_X∣D=0_ | | Exposure sensitivity for non-cases: The review of the vaccination records of N = 1000 individuals randomly selected from the study cohort (with vaccination coverage r = 75%) revealed four discrepancies (n = 4), all referring to subjects who had been vaccinated following the records review but were not recorded as such within the exposure data (12). Therefore, the false negative probability equals F_n_ = n/(r x N) = 4/(0.75 x 1000) = 0.005, implying that Se_X∣D=0_= 1 – F_n_ = 0.995 (mlik.) with min. = 0.986 and max. = 0.998 to account for uncertainty. | | | Βp(0.986,0.995,0.998) |  |
| Sp_X∣D=0_ | | Exposure specificity for non-cases: The review of the vaccination records of N = 1000 randomly selected individuals from the study cohort did not reveal false positives (12), implying that Sp_X∣D=0_ = 1. | | | I(1) |  |

S2 Derivation of prior distributions for the Monte Carlo based multiple-bias analyses, Finnish pediatric cohort-study (Nohynek, 2012).

Part C: Disease misclassification

| Parameter | | | Description: Rationale | Distribution |
| --- | --- | --- | --- | --- |
|  |  | |  |  |
| Disease misclassification | | | | |
|  | Se_D∣X=1_ | Disease sensitivity for vaccinated: Assuming an exponential distribution for time-to-diagnosis, the probability of diagnosis within t months of follow-up equals P = 1 – exp(-λt), with λ = 1/ μ being the inverse of the average time to diagnosis. Then, given t = 20 (observation period in the Finnish study) and given the observed average time-to-diagnosis intervals among the vaccinated based on the Swedish study (12 months; 17), Finnish study (7.9 months; 11), and French study (6.9 months; 15), SeD∣X=1 was calculated to be min. = 0.81, mlik.= 0.92 and max. = 0.95.  ALTERNATIVE: Assuming a baseline diagnostic delay of min.= 3, mlik.= 5 and max.= 10 years, assuming an exponential distribution for time-to-diagnosis and assuming that the probability of diagnosis doubled after public awareness, the probability of diagnosis within t = 20 months was calculated as above. | | Βp(0.81,0.92,0.95)  Βp(0.31,0.52,0.75) |
|  | Fr_D∣X=1_ | Number of false positive diagnoses per unit person-time (/100.000 py) among the vaccinated: False positives are assumed to happen only among the Brighton Collaboration (BC) level 3 cases. Given the proportion of BC level 3 cases (p_BC3_), given the false positive probability among BC level 3 cases (Fp_BC3_) and given the narcolepsy incidence (*inc*)*,* it follows that Fr_D∣X=1_= p_BC3_ x Fp_BC3_ x *inc*.  Given p_BC3_ = 8% and *inc* = 9/100.000 (5) and further assuming that Fp_BC3_ equals 5%, 35% and 50%, Fr_D∣X=1_ was calculated to be min.= 0.036, mlik.= 0.252 and max.= 0.36. | | Βp(0.036,0.252,0.36) |
|  | Se_D∣X=0_ | Disease sensitivity for unvaccinated: See also Se_D∣X=1_. Given t = 20 and given the observed average time-to-diagnosis intervals among the unvaccinated based on the Swedish study (60 months; 11), Finnish study (47.6 months; 5), and French study (12.6 months; 8), Se_D∣X=0_ was calculated to be min.= 0.28, mlik.= 0.34 and max.= 0.8.  ALTERNATIVE: Assuming a baseline diagnostic delay of min.= 3, mlik.= 5 and max.= 10years and assuming an exponential distribution for time-to-diagnosis, the probability of diagnosis within t = 20 months was calculated as above. | | Βp(0.28,0.34,0.8)  Βp(0.15,0.28,0.43) |
|  | Fr_D∣X=0_ | Number of false positive diagnosis per unit person-time (/100.000 py) among unvaccinated: See also Fr_D∣X=1_.  Given p_BC3_ = 8% and *inc* = 0.7/100.000 (12) and further assuming that Fp_BC3_ equals 5%, 15% and 50%, Fr_D∣X=0_ was calculated to be min.= 0.0028, mlik.= 0.0084 and max.= 0.028. | | Βp(0.0028,0.0084,0.028) |

S2 Derivation of prior distributions for the Monte Carlo based multiple-bias analyses, Finnish pediatric cohort-study (Nohynek, 2012).

Part D: Uncontrolled confounding: age group

| Parameter | | Description: Rationale | Distribution |
| --- | --- | --- | --- |
|  |  |  |  |
| Uncontrolled confounding: age group | | | |
|  | RR_CD(i)_ | Marginal association between age group (15-19years vs. 5-14years) and narcolepsy was obtained from a large European study on the incidence of narcolepsy (37). In particular, it was visually obtained from Figure 3: Pooled incidence of narcolepsy diagnosis (2000-10) by age and sex. (37). | Βp(2.4,3.3,4.6) |
|  | P_C∣X=1(i)_ | Prevalence of the age group 15-19years among vaccinated: The Finnish population size was N = 590209 for the 5-14 years old and N = 334636 for the 15-19 years old and the corresponding H1N1 pandemic vaccination coverage was 81% and 56% (16). Based on this information, the proportion of 15-19years olds among the vaccinated can be straightforwardly calculated. | I(0.28) |
|  | P_C∣X=0(i)_ | Prevalence of the age group 15-19years among unvaccinated is calculated using the same information as above | I(0.56) |

S2 Derivation of prior distributions for the Monte Carlo based multiple-bias analyses, Finnish pediatric cohort-study (Nohynek, 2012).

Part E: Uncontrolled confounding: risk group

| Parameter | | Description: Rationale | Distribution |
| --- | --- | --- | --- |
|  |  |  |  |
| Uncontrolled confounding: risk group | | | |
|  | RR_CD(ii)_ | Marginal association between ‘risk group’ and narcolepsy: In the UK study, 9% (p_risk_) of the population (2–18yrs) belonged to a clinical risk group, mainly because of asthma (16). The vaccine uptake in the ‘risk group’ (5-18yrs) was 27.1% compared to 1.9% in the non-risk group. The total population (5-18yrs) was 8,502,600 (N_tot_) of which 363,004 (N_vacc_) were vaccinated. Then, the probability of belonging to the ‘risk group’ among vaccinated was calculated as p_risk∣X=1_ = (27.1% x p_risk_ x N_tot_) / N_vacc_ = 57%. The probability of belonging to the ‘risk group’ among non-vaccinated was calculated as p_risk∣X=0_ = ((1-27.1%) x p_risk_ x N_tot_) / (N_tot_ - N_vacc_) = 6.9%. Furthermore, the vaccination-narcolepsy odds ratios were provided with matching on risk group (OR_adj_ = 14.4, 95% CI: 4.3 to 48.5) and without (OR_obs_ = 22.2, 95% CI: 7.9 to 62.1). Then, by rearranging Schlesselman’s formula (Schlesselman, *American Journal of Epidemiology*, 1978), equating odds ratios with and without correcting for uncontrolled confounding (9), the confounder-disease association can be obtained as  OR_CD_ = (OR_obs_ x (1- p_risk∣X=0_) – OR_adj_ x (1- p_risk∣X=1_)) /(OR_adj_ x p_risk∣X=1_ – OR_obs_ x p_risk∣X=0_).  Finally, by plugging-in the values above, the RR_CD_ was calculated using the mean, lower and upper estimates of OR_adj_ and OR_obs_, resulting in OR_CD(i)_ ≈ RR_CD(i)_ (rare disease) of min. = 1.58, mlik. = 2.16 and max. = 2.88. | Βp(1.56,2.11,2.8) |
|  | P_C∣X=1(ii)_ | Prevalence of ‘risk group’ among vaccinated: The total Finnish population (5-19yrs) was 924,845 (N_tot_), for which the vaccine uptake was 75% (p_vacc_). Then, given the proportion of the population belonging to the risk group (p_risk_) and given the vaccine uptake within that group (p_vacc∣C=1_), it follows that  P_C∣X=1(i)_ = (p_vacc∣C=1_ x p_risk_ x N_tot_)/(p_vacc_ x N_tot_).  Assuming that p_risk_ = 9% (as in the UK data; 16) and that p_vacc∣C=1_ equals at least the overall vaccination coverage within Finland (75%) as a result of the prioritization order of the vaccinations (12) (i.e. assuming that p_vacc∣C=1_ equals 75%, 90% and 100%), P_C∣X=1(i)_ was calculated as min. = 9%, mlik. = 11% and max. = 12%. | Βp(0.09,0.11,0.12) |
|  | P_C∣X=0(ii)_ | Prevalence of ‘risk group’ among unvaccinated: Analogous to P_C∣X=1(i)_, P_C∣X=0(i) =_ ((1-p_vacc∣C=1_) x p_risk_ x N_pop_)/((1-p_vacc_) x N_pop_).  Using the same information and assumptions as for P_C∣X=1(i)_, P_C∣X=0(i)_ was calculated as min. = 0%, mlik.= 4% and max.= 9%. | Βp(0,0.04,0.09) |

S2 Derivation of prior distributions for the Monte Carlo based multiple-bias analyses, Finnish pediatric cohort-study (Nohynek, 2012).

Part F: Uncontrolled confounding: natural H1N1 exposure

| Parameter | | Description: Rationale | Distribution |
| --- | --- | --- | --- |
|  |  |  |  |
| Uncontrolled confounding: natural H1N1 exposure | | | |
|  | RR_CD(iii)_ | Marginal association between H1N1 infection and narcolepsy: The average number of narcolepsy cases with onset outside the narcolepsy peaks (September to January, 1996-2010) was n_u_ = 25.5 and assumed to reflect the expected number of cases within the unexposed population (9). The number of cases within the narcolepsy peak following H1H1 epidemic was n_e_ = 143 and assumed to reflect the observed number of cases within a partially H1N1 exposed population (9). The amount of population exposure within that population (H1N1 attack rate) was p_exp_ = 31.8% (95%CI: 29.1-34.1) (38). Then, for a partially exposed population, the confounder-disease association can be obtained as  RR_CD_ = (n_e_ – (1 – p_exp_) x n_u_) /( p_exp_ x n_u_),  with the numerator referring to the observed and the denominator to the expected number of cases within the exposed part of the population. Then, RR_CD(ii)_ was calculated using the mean, lower and upper estimates of p_exp_, resulting in RR_CD(ii)_ of min.=14.5, mlik.=15.5 and max.=16.8. | Βp(14.9,16.4,17.5) |
|  | P_C∣X=1(iii)_ | Prevalence of H1N1 infection among vaccinated: Given the overall vaccination coverage (p_vacc_), the H1N1 attack rate (p_H1N1_) and the relative risk of vaccination given H1N1 exposure (RR_CX_), P_C∣X=1(ii)_ can be obtained as  P_C∣X=1(ii) =_ (p_H1N1_/p_vacc_ ) x RR_CX_ x ((1- p_vacc_)/p_vacc_ + RR_CX_ )^-1^  Given p_vacc_ = 75% (5) and assuming that (p_H1N1_) = 29% (33) and that RR_CX_ equals min. = 1, mlik. = 1.2 and max.= 1.5, P_C∣X=1(ii)_ was calculated as min. =29%, mlik. = 30% and max.= 32%. | Βp(0.29,0.3,0.32) |
|  | P_C∣X=0(iii)_ | Prevalence of H1N1 infection among unvaccinated: Given the overall vaccination coverage (p_vacc_), the H1N1 attack rate (p_H1N1_) and the relative risk of vaccination given H1N1 exposure (RR_CX_), P_C∣X=0(ii)_ can be obtained as  P_C∣X=0(ii) =_ (p_H1N1_/(1-p_vacc_)) x ((p_vacc_/(1-p_vacc_ )) x RR_CX_ + 1)^-1^  Using the same information and assumptions as for P_C∣X=1(ii)_, P_C∣X=0(ii)_ was calculated as min. = 21%, mlik.= 25% and max.= 29%. | Βp(0.21,0.25,0.29) |

S2 Derivation of prior distributions for the Monte Carlo based multiple-bias analyses, Finnish pediatric cohort-study (Nohynek, 2012).

Part G: Random error

| Parameter | | Description: Rationale | Distribution |
| --- | --- | --- | --- |
|  |  |  |  |
| Random error | | | |
|  | E | The log of the observed rate ratio is normally distributed with common maximum likelihood estimates of mean and variance. | N(0,1/n1 + 1/n0), with n1 = 46, n0 = 7 |

## References in tables S2 A-G are numbered according to the reference list in the full manuscript.
