# Supplementary material for: Application of Probabilistic Multiple-Bias Analyses to a Cohort- and a Case-Control Study on the Association between Pandemrix™and Narcolepsy
Source: PLoS One. 2016 Feb 22;11(2):e0149289. doi: 10.1371/journal.pone.0149289 (PMC4762678; doi:10.1371/journal.pone.0149289)
Supplement: S3 Table — (DOCX) [file pone.0149289.s003.docx]

S3 Derivation of prior distributions for the Monte Carlo based multiple-bias analyses, French case-control study (Dauvilliers, 2013).

Part A: Observed data.

| Parameter | | **Description: Rationale** | **Distribution** |
| --- | --- | --- | --- |
|  |  |  |  |
| Observed data | | | |
|  | a | Number of vaccinated cases. | 31 |
|  | b | Number of unvaccinated cases. | 28 |
|  | c | Number of vaccinated controls. | 24 |
|  | d | Number of unvaccinated controls. | 111 |

S3 Derivation of prior distributions for the Monte Carlo based multiple-bias analyses, French case-control study (Dauvilliers, 2013).

Part B: Exposure misclassification.

| Parameter | **Description: Rationale** | | **Distribution** | |  |
| --- | --- | --- | --- | --- | --- |
|  | |  | |  | |
| Exposure misclassification | | | | |  |
| Se_X\|D=1_ | | Exposure sensitivity for cases: Medical records of cases are scrutinized | | I(1) | |
| Sp_X\|D=1_ | | Exposure specificity for cases: Vaccination could be documented for all cases (15). | | I(1) | |
| Se_X\|D=0_ | | Exposure sensitivity for controls: In studies comparing self-reported influenza vaccination status to vaccination records, reported exposure sensitivities with an average value of 96% were reported when considering risk groups and health care workers (42). In France, a special procedure was implemented for H1N1 vaccination. Therefore, the sensitivity was assumed to be higher. | | Βp(0.97,0.98,1) | |
| Sp_X∣D=0_ | | Exposure specificity for controls: Vaccination was not documented for 6 controls. If x of them are false positive, it follows that  Sp_X∣D=0_ = 1 – x/(d+x)  Given c =24 and d = 111 and further assuming that x equals 0, 3 and 6, Sp_X∣D=0_ was calculated to be min. = 0.95, mlik. = 0.97 and max.=1. | | Βp(0.95,0.97,1) | |

S3 Derivation of prior distributions for the Monte Carlo based multiple-bias analyses, French case-control study (Dauvilliers, 2013).

Part C: Disease misclassification.

| Parameter | | **Description: Rationale** | **Distribution** |
| --- | --- | --- | --- |
|  |  |  |  |
| Disease misclassification | | | |
|  | Se_D∣X=1_ | Disease sensitivity for vaccinated: Only narcolepsy –cataplexy cases (BC level 1 and level 2) cases were selected. Therefore, perfect disease sensitivity and specificity was assumed. | I(1) |
|  | Sp_D∣X=1_ | Disease specificity for vaccinated: See also Se_D∣X=1_. | I(1) |
|  | Se_D∣X=0_ | Disease sensitivity for unvaccinated: See also Se_D∣X=1_. | I(1) |
|  | Sp_D∣X=0_ | Disease specificity for unvaccinated: See also Se_D∣X=1_. | I(1) |

S3 Derivation of prior distributions for the Monte Carlo based multiple-bias analyses, French case-control study (Dauvilliers, 2013).

Part D: Selection bias.

| Parameter | | **Description: Rationale** | **Distribution** |
| --- | --- | --- | --- |
|  |  |  |  |
| Selection bias | | | |
|  | P_case∣X=1_ | Selection probability of a vaccinated case: (a) Differential ascertainment and (b) participation bias independently affect the selection probability of a case. Therefore, P_case∣X=1_= P_case(a)∣X=1_ x P_case(b)∣X=1._  (a) Differential ascertainment: Assuming an exponential distribution for time-to-diagnosis, the probability of diagnosis within *t* months of follow-up equals P = 1 – exp(-λ*t*), with λ = 1/ μ being the inverse of the average time to diagnosis. Then, given t = 19 (recruitment period of the French study) and given the observed average time-to-diagnosis intervals among the exposed based on the Swedish study (12 months; 17), Finnish study (7.9 months; 11), and French study (6.9 months; 15), P_case(a)∣X=1_ was calculated to be min. = 0.79, mlik.= 0.91 and max. = 0.94.  ALTERNATIVE: Assuming a baseline diagnostic delay of min.= 3, mlik.= 5 and max.= 10 years, assuming an exponential distribution for time-to-diagnosis and assuming that the probability of diagnosis doubled after public awareness, the probability of diagnosis within t = 19 months was calculated as above.  (b) Participation bias: Given the number of participating cases *n_1_* and the corresponding participation rate *r*, the number of non-participating cases *n_0_* is obtained as *n_0_* = *n_1_*(1/*r* – 1). Then, given the vaccination probability among the non-participating cases (p_vacc∣N0_), the selection probability of an exposed case reflecting participation bias can be obtained as  P_case(b)∣X=1_= a/(p_vacc∣N0_ x *n_0_* +a)  Given a = 31 and r = 0.71 and further assuming that p_vacc∣N0_ equals 0.3, 0.45 and 0.52 (equals the observed exposure prevalence among the participating cases, implying the assumption of no participation bias), P_case(b)∣X=1_ was calculated as min. = 0.71, mlik. = 0.74 and max. = 0.81. | Βp(0.79,0.90,0.94)  Βp(0.15,0.27,0.41)  Βp(0.71,0.74,0.81) |
|  | P_case∣X=0_ | Selection probability of an unvaccinated case: (a) Differential ascertainment and (b) participation bias independently affect the selection probability of a case. Therefore, P_case∣X=0_ = P_case(a)∣X=0_ x P_case(b)∣X=0._  (a) Differential ascertainment: See also P_case∣X=1_. Given t = 19 and given the observed average time-to-diagnosis intervals among the unvaccinated based on the Swedish study (60 months; 17), Finnish study (47.6 months; 11) and French study (12.6 months; 15), P_case(a)∣X=0_ was calculated as min.= 0.27, mlik.= 0.33 and max.= 0.78.  ALTERNATIVE: Assuming a baseline diagnostic delay of min.= 3, mlik.= 5 and max.= 10years and assuming an exponential distribution for time-to-diagnosis, the probability of diagnosis within t = 19 months was calculated as above.  (b) Participation bias: The selection probability of an unvaccinated case due to participation bias is determined by this of the vaccinated case (see P_case∣X=1_). Let N be the total number of participating cases or N = a+b and r the participating rate, then N/r is total number of eligible cases and the selection probability of an unvaccinated case reflecting participation bias is  P_case(b)∣X=0_ = b/(N/r - a/P_case(b)∣X=1_). | Βp(0.27,0.33,0.78)  Βp(0.30,0.51,0.73)  = b/(N/r - a/P_case(b)∣X=1_) |
|  | P_control∣X=1_ | Selection probability of a vaccinated control: The selection probabilities of vaccinated and unvaccinated controls were judged to be disproportional because (i) hospital controls were recruited from departments that specialized in treatment of patients for which H1N1-vaccination was not recommended and (ii) healthy controls were recruited from NARCOBANK, among which many health care workers for which vaccination was recommended. Hence, the selection probability is unlikely to be independent of exposure with bias in either direction. Therefore, the disproportionality in selection probabilities, λ=P_control∣X=0_ /P_control∣X=1_, was assumed to be min. = 0.8, mlik.= 1 and max. = 1.2. | = λ x P_control∣X=0_ , with  λ ∼ Βp(0.8,1,1.2) |
|  | P_control∣X=0_ | Selection probability of an unvaccinated control: Because only the disproportionality of the sampling weights induces selection bias, P_control∣X=0_ can take any constant value. | I(C^t^) |

S2 Derivation of prior distributions for the Monte Carlo based multiple-bias analyses, Finnish pediatric cohort-study (Nohynek, 2012).

Part E: Uncontrolled confounding: age group

| Parameter | | Description: Rationale | Distribution |
| --- | --- | --- | --- |
|  |  |  |  |
| Uncontrolled confounding: age group | | | |
|  | RR_CD(i)_ | Marginal association between age group (18-29 years vs. 5-17 years) and narcolepsy was obtained from a large European study on the incidence of narcolepsy (37). In particular, it was visually obtained from Figure 3: Pooled incidence of narcolepsy diagnosis (2000-10) by age and sex. (37). | Βp(1.3,1.45,1.6) |
|  | P_C∣X=1(i)_ | Prevalence of the age group 18-29 years among vaccinated: The French population size was N = 10302212 for the 5-17 years old, N = 9758498 for the 18-29 years old and N = 18369480 for the 30-50 years old (INSEE, population by age on 1^st^ Jan 2009) and the corresponding H1N1 pandemic vaccination coverage was +- 10%, 4% and 7.5% (43). Based on this information, the proportion of 18-29 years olds among the vaccinated can be straightforwardly calculated. | Βp(0.13,0.15,0.17) |
|  | P_C∣X=0(i)_ | Prevalence of the age group 18-29 years among unvaccinated is calculated using the same information as above | Βp(0.22,0.24,0.26) |
|  | RR_CD(i)_ | Marginal association between age group (30-50yrs vs. 5-17yrs) and narcolepsy was obtained as above | Βp(0.96,1.08,1.23) |
|  | P_C∣X=1(i)_ | Prevalence of the age group 30-50 years among vaccinated is calculated using the same information as above | Βp(0.45,0.47,0.49) |
|  | P_C∣X=0(i)_ | Prevalence of the age group 30-50 years among unvaccinated is calculated using the same information as above | Βp(0.42,0,44,0.46) |

S3 Derivation of prior distributions for the Monte Carlo based multiple-bias analyses, French case-control study (Dauvilliers, 2013).

Part F: uncontrolled confounding: risk group.

| Parameter | | **Description: Rationale** | **Distribution** |
| --- | --- | --- | --- |
|  |  |  |  |
| uncontrolled confounding: risk group | | | |
|  | RR_CD(i)_ | Marginal association between ‘risk group’ and narcolepsy: In the UK study, 9% (p_risk_) of the population (2–18yrs) belonged to a clinical risk group, mainly because of asthma (16). The vaccine uptake in the ‘risk group’ (5-18yrs) was 27.1% compared to 1.9% in the non-risk group. The total population (5-18yrs) was 8,502,600 (N_tot_) of which 363,004 (N_vacc_) were vaccinated. Then, the probability of belonging to the ‘risk group’ among vaccinated was calculated as p_risk∣X=1_ = (27.1% x p_risk_ x N_tot_) / N_vacc_ = 57%. The probability of belonging to the ‘risk group’ among non-vaccinated was calculated as p_risk∣X=0_ = ((1-27.1%) x p_risk_ x N_tot_) / (N_tot_ - N_vacc_) = 6.9%. Furthermore, the vaccination-narcolepsy odds ratios were provided with matching on risk group (OR_adj_ = 14.4, 95% CI: 4.3 to 48.5) and without (OR_obs_ = 22.2, 95% CI: 7.9 to 62.1). Then, by rearranging Schlesselman’s formula (Schlesselman, *American Journal of Epidemiology*, 1978), equating odds ratios with and without correcting for uncontrolled confounding (9), the confounder-disease association can be obtained as  OR_CD_ = (OR_obs_ x (1- p_risk∣X=0_) – OR_adj_ x (1- p_risk∣X=1_)) /(OR_adj_ x p_risk∣X=1_ – OR_obs_ x p_risk∣X=0_).  Finally, by plugging-in the values above, the RR_CD_ was calculated using the mean, lower and upper estimates of OR_adj_ and OR_obs_, resulting in OR_CD(i)_ ≈ RR_CD(i)_ (rare disease) of min. = 1.56, mlik. = 2.11 and max. = 2.8. | Βp(1.56,2.11,2.8) |
|  | P_C∣X=1(i)_ | Prevalence of ‘risk group’ among vaccinated: Based on vaccine uptake study in France (44), the total vaccination coverage was estimated as p_cov_ = 12.8% (N = 1485) and the coverage within the risk group as p_cov∣C=1_ = 21% (95%CI: 15%-27%; n = 184). Then, P_C∣X=1(i)_ can be obtained as  P_C∣X=1(i)_ = (p_cov∣C=1_ x n)/(p_cov_ x N).  Using the mean, lower and upper estimates of p_cov∣C=1_, P_C∣X=1(i)_ was calculated as min.= 15%, mlik. = 21% and max.= 26%. | Βp(0.15,0.21,0.26) |
|  | P_C∣X=0(i)_ | Prevalence of ‘risk group’ among unvaccinated: See also P_C∣X=1(i)_. Using the same estimates of the vaccination coverages as above, P_C∣X=0(i)_ can be obtained as  P_C∣X=0(i)_ = ((1-p_cov∣C=1_) x n)/((1-p_cov_) x N).  Using the mean, lower and upper estimates of p_cov∣C=1_, P_C∣X=0(i)_ was calculated as min.= 10%, mlik. = 11% and max.= 12%. | Βp(0.1,0.11,0.12) |
|  |  |  |  |

S3 Derivation of prior distributions for the Monte Carlo based multiple-bias analyses, French case-control study (Dauvilliers, 2013).

Part G: uncontrolled confounding: natural H1N1 exposure.

| Parameter | | **Description: Rationale** | **Distribution** |
| --- | --- | --- | --- |
|  |  |  |  |
| uncontrolled confounding: natural H1N1 exposure | | | |
|  | RR_CD(ii)_ | Marginal association between H1N1 infection and narcolepsy: The average number of narcolepsy cases with onset outside the narcolepsy peaks (September to January, 1996-2010) was n_u_ = 25.5 and assumed to reflect the expected number of cases within the unexposed population (9). The number of cases within the narcolepsy peak following H1H1 epidemic was n_e_ = 143 and assumed to reflect the observed number of cases within a partially H1N1 exposed population (9). The amount of population exposure within that population (H1N1 attack rate) was p_exp_ = 31.8% (95%CI: 29.1-34.1) (38). Then, for a partially exposed population, the confounder-disease association can be obtained as  RR_CD_ = (n_e_ – (1 – p_exp_) x n_u_) /( p_exp_ x n_u_),  with the nominator referring to the observed and the denominator to the expected number of cases within the exposed part of the population. Then, RR_CD(ii)_ was calculated using the mean, lower and upper estimates of p_exp_, resulting in RR_CD(ii)_ of min.=14.9, mlik.=16.4 and max.=17.5. | Βp(14.9,16.4,17.5) |
|  | P_C∣X=1(ii)_ | Prevalence of H1N1 infection among vaccinated: Given the overall vaccination coverage (p_vacc_), the H1N1 attack rate (p_H1N1_) and the relative risk of vaccination given H1N1 exposure (RR_CX_), P_C∣X=1(ii)_ can be obtained as  P_C∣X=1(ii) =_ (p_H1N1_/p_vacc_ ) x RR_CX_ x ((1- p_vacc_)/p_vacc_ + RR_CX_ )^-1^  Given p_vacc_ = 8.8% (15) and assuming that (p_H1N1_) = 29% (39) and that RR_CX_ equals min. = 1, mlik. = 1.2 and max. = 1.5, P_C∣X=1(ii)_ was calculated as min. =29%, mlik. = 34% and max.= 42%. | Βp(0.29,0.34,0.42) |
|  | P_C∣X=0(ii)_ | Prevalence of H1N1 infection among unvaccinated: Given the overall vaccination coverage (p_vacc_), the H1N1 attack rate (p_H1N1_) and the relative risk of vaccination given H1N1 exposure (RR_CX_), P_C∣X=0(ii)_ can be obtained as  P_C∣X=0(ii) =_ (p_H1N1_/(1-p_vacc_)) x ((p_vacc_/(1-p_vacc_ )) x RR_CX_ + 1)^-1^  Using the same information and assumptions as for P_C∣X=1(ii)_, P_C∣X=0(ii)_ was calculated as min. = 28%, mlik.= 28.5% and max.= 29%. | Βp(0.28,0.285,0.29) |

S3 Derivation of prior distributions for the Monte Carlo based multiple-bias analyses, French case-control study (Dauvilliers, 2013).

Part H: Random error.

| Parameter | | **Description: Rationale** | **Distribution** |
| --- | --- | --- | --- |
|  |  |  |  |
| Random error | | | |
|  | E | The log of the observed odds ratio is normally distributed with common maximum likelihood estimates of mean and variance. | N(0,1/a + 1/b + 1/c + 1/d) with a = 31, b = 28, c= 24 and d = 111 |

## References in tables S3 A-H are numbered according to the reference list in the full manuscript.
