# Supplementary material for: Application of Probabilistic Multiple-Bias Analyses to a Cohort- and a Case-Control Study on the Association between Pandemrix™and Narcolepsy
Source: PLoS One. 2016 Feb 22;11(2):e0149289. doi: 10.1371/journal.pone.0149289 (PMC4762678; doi:10.1371/journal.pone.0149289)
Supplement: S4 Table — (DOCX) [file pone.0149289.s004.docx]

S4. Example Code Multiple-bias Analysis.

Cohort study

### EXAMPLE CODE – uncorrelated values

### use R package (to randomly sample from betapert (‘rpert’) distribution) ###

library("mc2d")

### OBSERVED DATA Nohynek (PlosOne,2012): ###

n1 <- 46 # number of exposed cases

n0 <- 7 # number of unexposed cases

t1 <- 510874 # follow-up time (person years) among exposed

t0 <- 986195 # follow-up time (person years) among unexposed

py <- 100000 # unit person time (RR per 100,000 person years)

### RANDOMLY SAMPLE from input distributions ###

### (fast computation through vectorization) ###

set.seed(123454321) # number to initialize pseudorandom number generator

K <- 500000 # number of runs

## Exposure misclassification ##

# (1.1) exposure sensitivity for cases

Se_X1 <- rep(1,K)

# (1.2) exposure specificity for cases

Sp_X1 <- rep(1,K)

# (1.3) exposure sensitivity for non-cases

Se_X0 <- rpert(K, min=0.986, mode=0.995, max = 0.998, shape = 4)

# (1.4) exposure specificity for non-cases

Sp_X0 <- rep(1,K)

## Disease misclassification ##

# (2.1) disease sensitivity for exposed

Se_D1 <- rpert(K, min=0.81, mode=0.92, max = 0.95, shape = 4)

# (2.2) number of false positives per unit person time (/100000 py) among exposed

Fr_D1 <- rpert(K, min=0.036, mode=0.252, max = 0.36, shape = 4)

# (2.3) disease sensitivity for unexposed

Se_D0 <- rpert(K, min=0.28, mode=0.34, max = 0.8, shape = 4)

# (2.4) number of false positives per unit person time (/100000 py) among unexposed

Fr_D0 <- rpert(K, min=0.0028, mode=0.0084, max = 0.028, shape = 4)

## Confounding by age group ##

# (3.1) marginal association confounder-disease

RR_CDa <- rpert(K, min=2.4, mode=3.3, max = 4.6, shape = 4)

# (3.2) prevalence confounder among exposed

P_C1a <- rep(0.28,K)

# (3.3) prevalence confounder among unexposed

P_C0a <- rep(0.56, K)

## Confounding by risk group ##

# (4.1) marginal association confounder-disease

RR_CDb <- rpert(K, min=1.56, mode=2.11, max = 2.8, shape = 4)

# (4.2) prevalence confounder among exposed

P_C1b <- rpert(K, min=0.09, mode=0.11, max = 0.12, shape = 4)

# (4.3) prevalence confounder among unexposed

P_C0b <- rpert(K, min=0, mode=0.04, max = 0.09, shape = 4)

## Confounding by natural H1N1 infection ##

# (5.1) marginal association confounder-disease

RR_CDc <- rpert(K, min=14.5, mode=15.5, max = 16.8, shape = 4)

# (5.2) prevalence confounder among exposed

P_C1c <- rpert(K, min=0.29, mode=0.30, max = 0.32, shape = 4) Ò

# (5.3) prevalence confounder among unexposed

P_C0c <- rpert(K, min=0.21, mode=0.25, max = 0.29, shape = 4)

## random error ##

lnRR_se <- sqrt(1/n1 + 1/n0)

e <- rnorm(K,mean=0,sd=lnRR_se)

### SEQUENCE OF BIAS CORRECTIONS ###

## Exposure misclassification ##

# number of exposed cases 'adjusted' for exposure misclassification

s1.n1 <- ((Sp_X1*n1) - ((1 - Sp_X1)*n0)) / ((Se_X1*Sp_X1) - ((1 - Se_X1) *(1 - Sp_X1)))

# number of unexposed cases 'adjusted' for exposure misclassification

s1.n0 <- (n1 + n0) - s1.n1

# follow-up time among exposed 'adjusted' for exposure misclassification

s1.t1 <- ((Sp_X0*t1) - ((1 - Sp_X0)*t0)) / ((Se_X0*Sp_X0) - ((1 - Se_X0)*(1 - Sp_X0)))

# follow-up time among unexposed 'adjusted' for exposure misclassification

s1.t0 <- (t1 + t0) - s1.t1

# RR 'adjusted' for exposure misclassification

s1.RR <- (s1.n1/s1.t1)/(s1.n0/s1.t0)

## Disease misclassification ##

# number of exposed cases 'adjusted' for exposure- and disease misclassification

s2.n1 <- (s1.n1 - (Fr_D1*(s1.t1/py)))/Se_D1

# number of unexposed cases 'adjusted' for exposure- and disease misclassification

s2.t1 <- s1.t1

# follow-up time among exposed 'adjusted' for exposure- and disease misclassification

s2.n0 <- (s1.n0 - (Fr_D0*(s1.t0/py)))/Se_D0

# follow-up time among unexposed 'adjusted' for exposure- and disease misclassification

s2.t0 <- s1.t0

# RR 'adjusted' for exposure- and disease misclassification

s2.RR <- (s2.n1/s2.t1)/(s2.n0/s2.t0)

## Unmeasured confounding - age group ##

# RR 'adjusted' for exposure-, disease misclassification and confounding by age group

s3.RR = s2.RR * (RR_CDa*P_C0a + (1-P_C0a)) / (RR_CDa*P_C1a + (1-P_C1a))

## Unmeasured confounding - risk group ##

# RR 'adjusted' for exposure-, disease misclassification and confounding by age group and risk group

s4.RR = s3.RR * (RR_CDb*P_C0b + (1-P_C0b)) / (RR_CDb*P_C1b + (1-P_C1b))

## Unmeasured confounding - H1N1 exposure ##

# RR 'adjusted' for exposure-, disease misclassification and confounding by age group, risk group and H1N1 exposure

s5.RR = s4.RR * (RR_CDc*P_C0c + (1-P_C0c)) / (RR_CDc*P_C1c + (1-P_C1c))

## Random error ##

s6.RR <- exp(log(s5.RR) + e)

### SUMMARIZE results ###

# median

med <- round(median(s6.RR), digits = 2)

# lower and upper bound of percentile-based 95% Confidence Interval

lower <- round(quantile(s6.RR,probs=c(0.025)), digits=2)

upper <- round(quantile(s6.RR,probs=c(0.975)), digits=2)

Case-control study

# EXAMPLE CODE – uncorrelated values

### use R package (to randomly sample from betapert (‘rpert’) distribution) ###

library("mc2d")

### OBSERVED DATA Dauvilliers (Brain,2013): ###

a <- 31 # number of exposed cases

b <- 28 # number of unexposed cases

c <- 24 # number of exposed controls

d <- 111 # number of unexposed controls

### RANDOMLY SAMPLE from input distributions ###

### (fast computation through vectorization) ###

set.seed(123454321) # number to initialize pseudorandom number generator

K <- 500000 # number of runs

## Exposure misclassification ##

# (1.1) exposure sensitivity for cases

Se_X1 <- rep(1,K)

# (1.2) exposure specificity for cases

Sp_X1 <- rep(1,K)

# (1.3) exposure sensitivity for non-cases

Se_X0 <- rpert(K, min=0.97, mode=0.98, max=1, shape = 4)

# (1.4) exposure specificity for non-cases

Sp_X0 <- rpert(K, min=0.95, mode=0.97, max=1, shape = 4)

## Disease misclassification ##

# (2.1) disease sensitivity for exposed

Se_D1 <- rep(1,K)

# (2.2) disease specificity for exposed

Sp_D1 <- rep(1,K)

# (2.3) disease sensitivity for unexposed

Se_D0 <- rep(1,K)

# (2.4) disease specificity for unexposed

Sp_D0 <- rep(1,K)

## Selection bias ##

# (3.1) selection probability of exposed case

Pcase_1a <- rpert(K, min=0.79, mode=0.9, max=0.94, shape = 4)

Pcase_1b <- rpert(K, min=0.71, mode=0.74, max=0.81, shape = 4)

Pcase_1 <- Pcase_1a * Pcase_1b

# (3.2) selection probability of unexposed case

Pcase_0a <- rpert(K, min=0.27, mode=0.33, max = 0.78, shape = 4)

Pcase_0b <- b/((a+b)/0.71-a/Pcase_1b)

Pcase_0 <- Pcase_0a * Pcase_0b

# (3.3) selection probability of unexposed control

Pcontrol_0 <- rep(0.5,K)

# (3.4) selection probability of exposed control

Lambda <- rpert(K, min=0.8, mode=1, max = 1.2, shape = 4)

Pcontrol_1 <- Pcontrol_0 * Lambda

## Confounding by age (polytomous confounder) ##

# (4.1) marginal association confounder-disease – age group 18-29yrs vs 5-17yrs

OR_Cda_1 <- rpert(K, min=1.3, mode=1.45, max = 1.6, shape = 4)

# (4.2) prevalence confounder among exposed

P_C1a_1 <- rpert(K, min=0.13, mode=0.15, max = 0.17, shape = 4)

# (4.3) prevalence confounder among unexposed

P_C0a_1 <- rpert(K, min=0.22, mode=0.24, max = 0.26, shape = 4)

# (4.4) marginal association confounder-disease – age group 30-50yrs vs 5-17yrs

OR_Cda_2 <- rpert(K, min=0.96, mode=1.08, max = 1.23, shape = 4)

# (4.5) prevalence confounder among exposed

P_C1a_2 <- rpert(K, min=0.45, mode=0.47, max = 0.49, shape = 4)

# (4.6) prevalence confounder among unexposed

P_C0a_2 <- rpert(K, min=0.42, mode=0.44, max = 0.46, shape = 4)

## Confounding by risk group ##

# (5.1) marginal association confounder-disease

OR_CDb <- rpert(K, min=1.56, mode=2.11, max = 2.8, shape = 4)

# (5.2) prevalence confounder among exposed

P_C1b <- rpert(K, min=0.15, mode=0.21, max = 0.26, shape = 4)

# (5.3) prevalence confounder among unexposed

P_C0b <- rpert(K, min=0.1, mode=0.11, max = 0.12, shape = 4)

## Confounding by natural H1N1 infection ##

# (6.1) marginal association confounder-disease

OR_CDc <- rpert(K, min=14.9, mode=16.4, max = 17.5, shape = 4)

# (6.2) prevalence confounder among exposed

P_C1c <- rpert(K, min=0.29, mode=0.34, max = 0.42, shape = 4)

# (6.3) prevalence confounder among unexposed

P_C0c <- rpert(K, min=0.28, mode=0.285, max = 0.29, shape = 4)

## random error ##

lnOR_se <- sqrt(1/a + 1/b + 1/c + 1/d)

e <- rnorm(K,mean=0,sd=lnOR_se)

### SEQUENCE OF BIAS CORRECTIONS ###

## Exposure misclassification ##

# number of exposed cases 'adjusted' for exposure misclassification

s1.a <- ((Sp_X1*a) - ((1-Sp_X1)*b)) / ((Se_X1*Sp_X1) - ((1-Se_X1) *(1-Sp_X1)))

# number of unexposed cases 'adjusted' for exposure misclassification

s1.b <- (a + b) - s1.a

# number of exposed controls 'adjusted' for exposure misclassification

s1.c <- ((Sp_X0*c) - ((1-Sp_X0)*d)) / ((Se_X0*Sp_X0) - ((1-Se_X0) *(1-Sp_X0)))

# number of unexposed controls 'adjusted' for exposure misclassification

s1.d <- (c + d) - s1.c

# OR 'adjusted' for exposure misclassification

s1.OR <- (s1.a*s1.d)/(s1.b*s1.c)

## Disease misclassification ##

# number of exposed cases 'adjusted' for exposure- and disease misclassification

s2.a <- (s1.a - (s1.a + s1.c)*(1-Sp_D1))/(Se_D1 - (1-Sp_D1))

# number of unexposed cases 'adjusted' for exposure- and disease misclassification

s2.b <- (s1.b - (s1.b + s1.d)*(1-Sp_D0))/(Se_D0 - (1-Sp_D0))

# number of exposed controls ‘adjusted’ for exposure- and disease misclassification

s2.c <- (s1.a + s1.c) - s2.a

# number of unexposed controls ‘adjusted’ for exposure- and disease misclassification

s2.d <- (s1.b + s1.d) - s2.b

# OR 'adjusted' for exposure- and disease misclassification

s2.OR <- (s2.a*s2.d)/(s2.b*s2.c)

## Selection bias ##

#nr of exposed cases 'adjusted' for exposure-disease misclassification and selection

s3.a <- s2.a / Pcase_1

# nr of unexposed cases 'adjusted' for exposure-disease misclassification and selection

s3.b <- s2.b / Pcase_0

#nr of exposed controls ‘adjusted’ for exposure-disease misclassification and selection

s3.c <- s2.c / Pcontrol_1

#nr of unexposed controls ‘adjusted’ for exposure-disease misclassification and selection

s3.d <- s2.d / Pcontrol_0

# OR 'adjusted' for exposure- and disease misclassification

s3.OR <- (s3.a*s3.d)/(s3.b*s3.c)

## Unmeasured confounding - age groups (polytomous) ##

# OR 'adjusted' for exposure-, disease misclassification and confounding by age group

dd <- (OR_Cda_2 * p_C0a_2) + (OR_Cda_1 * p_C0a_1) + (1 - p_C0a_1 - p_C0a_2)

nn <- (OR_Cda_2 * p_C1a_2) + (OR_Cda_1 * p_C1a_1) + (1 - p_C1a_1 - p_C1a_2)

s4.OR = s3.OR * (dd/nn)

## Unmeasured confounding - risk group ##

# OR 'adjusted' for exposure-, disease misclassification and confounding by age and risk group

s5.OR = s4.OR * (OR_CDb*P_C0b + (1-P_C0b)) / (OR_CDb*P_C1b + (1-P_C1b))

## Unmeasured confounding - H1N1 exposure ##

# OR 'adjusted' for exposure-, disease misclassification and confounding by age group, risk group and H1N1 exposure

s6.OR = s5.OR * (OR_CDb*P_C0c + (1-P_C0c)) / (OR_CDb*P_C1c + (1-P_C1c))

## Random error ##

s7.OR <- exp(log(s6.OR) + e)

### SUMMARIZE results ###

# median

med <- round(median(s7.OR), digits=2)

# lower and upper bound of percentile-based 95% Confidence Interval

lower <- round(quantile(s7.OR,probs=c(0.025)), digits=2)

upper <- round(quantile(s7.OR,probs=c(0.975)), digits=2)

Sampling correlated values using Gaussian copulas

require(mvtnorm)

corr = 0.95 #set correlation

S <- matrix(c(1,corr,corr,1),2,2) #build correlation matrix

AB <- rmvnorm(mean=c(0,0),sig=S,n=K) #randomly sample from multivariate normal

U <- pnorm(AB) #transform using cumulative distribution function

# randomly sample correlated values from betapert distributions

P1 <- qpert(U[,1], min=0.1, mode=0.2, max = 0.3, shape = 4)

P1 <- qpert(U[,2], min=0.4, mode=0.5, max = 0.6, shape = 4)
